# Heterogeneous integrated dataset for Maritime Intelligence, surveillance, and reconnaissance

**DOI:** 10.1016/j.dib.2019.104141

**Published:** 2019-06-11

**Authors:** Cyril Ray, Richard Dréo, Elena Camossi, Anne-Laure Jousselme, Clément Iphar

**Affiliations:** a Naval Academy Research Institute, Brest, France; b Arts et Métiers ParisTech, France; c NATO STO Centre for Maritime Research and Experimentation (CMRE), La Spezia, Italy

## Abstract

Facing an ever-increasing amount of traffic at sea, many research centres, international organisations, and industrials have favoured and developed sensors together with detection techniques for the monitoring, analysis, and visualisation of sea movements. The Automatic Identification System (AIS) is one of the electronic systems that enable ships to broadcast their position and nominative information via radio communication. In addition to these systems, the understanding of maritime activities and their impact on the environment also requires contextual maritime data capturing additional features to ships' kinematic from complementary data sources (environmental, contextual, geographical, …). The dataset described in this paper contains ship information collected through the AIS, prepared together with spatially and temporally correlated data characterising the vessels, the area where they navigate and the situation at sea. The dataset contains four categories of data: navigation data, vessel-oriented data, geographic data, and environmental data. It covers a time span of six months, from October 1st, 2015 to March 31st, 2016 and provides ship positions over the Celtic sea, the North Atlantic Ocean, the English Channel, and the Bay of Biscay (France). The dataset is proposed for an easy integration with relational databases. This relies on the widespread and open source relational database management system PostgreSQL, with the adjunction of the geospatial extension PostGIS for the treatment of all spatial features of the dataset.

**Table:** Specifications Table

Subject area	*Maritime Mobility and Transport*
More specific subject area	*Intelligence, Surveillance, and Reconnaissance for Fisheries*
Type of data	*Position of ships, geographical, contextual and environmental data related to maritime navigation*
How data was acquired	*The dataset was created by combing data collected with a terrestrial receiver of the Automatic Identification System (Class A SAAB R4) and publicly available nominative, geographical and environmental datasets*
Data format	*Comma-separated values (CSV), ESRI shapefile*
Experimental factors	*Spatial, temporal and message-based filtering of received data*
Experimental features	*Automatic Identification System data collection and pre-processing, Guided search of open source maritime data on the web, Identification and reporting of linked nautical charts.*
Data source location	*Celtic sea, North Atlantic ocean, English Channel, Bay of Biscay (France)*
Data accessibility	*Heterogeneous Integrated Dataset for Maritime Intelligence, Surveillance, and Reconnaissance (HIDMISR)*[3] *Identifier:*https://doi.org/10.5281/zenodo*.1167594* *Usage rights: Creative Commons Attribution-NonCommercial-ShareAlike 4.0 International (CC BY-NC-SA 4.0)*

**Table:** 

**Value of the data** The dataset is realistic and comprehensive, **representative of operational information needs** in different Maritime Intelligence, Surveillance, and Reconnaissance scenarios, including safety and security of navigation and preservation of protected areas. It will suit various researches and potential applications and reuses, including: • **Maritime traffic analysis**: The AIS data volume is large enough to support the development of sea traffic models to characterise the maritime traffic and for estimating other *patterns of life.* • **Impact assessment of human activities at sea**: The dataset may provide a valuable support for understanding the impact of human activities at sea, facilitating the development of sustainable fishery policies (e.g., by developing fishing pressure models^1^). • **Environmental assessment**: It might be beneficial to ocean dynamics estimation study, to assess the quality of specific ocean state variables (e.g., currents). • **Assessment and benchmarking of multi-source information fusion algorithms**: The dataset gathers highly heterogeneous data, mixing raw and processed information, archival data and streaming data. Since these heterogeneous data are aligned in time and space, they are a perfect test bed to experiment multi-source information fusion algorithms. In addition, the dataset is becoming a **reference benchmark AIS dataset**, profitable also for maritime information training activities, because the included AIS data are of high quality and have been validated, and being a mix of proprietary and public datasets, it would contribute to increase the value of **public institutional data**.

## 1 Data

The dataset described in this paper contains ship information collected through the Automatic Identification System (AIS), validated and integrated with correlated contextual data characterising the vessels, the area where they are navigating and the concomitant sea conditions. It extends over six months, from October 1st, 2015 to March 31st, 2016 and covers the Celtic sea, the North Atlantic Ocean, the English Channel and Bay of Biscay (France).

**Fig. 1:**
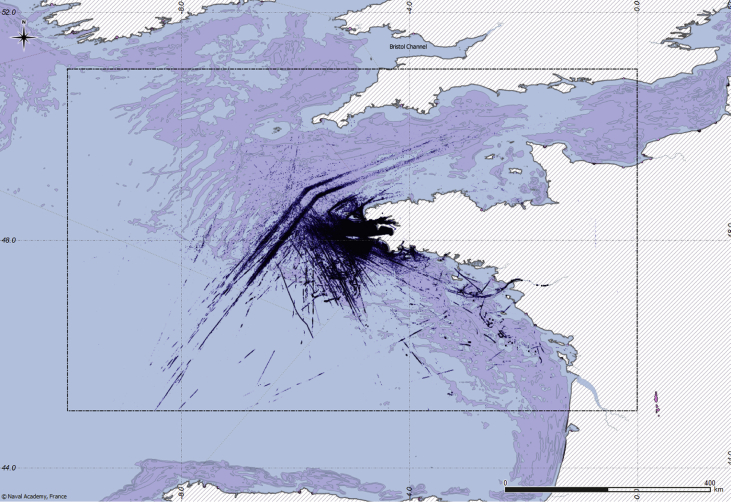
Navigation data (*purple background represents fishing areas computed by* Ref. [2]).

**Fig. 2:**
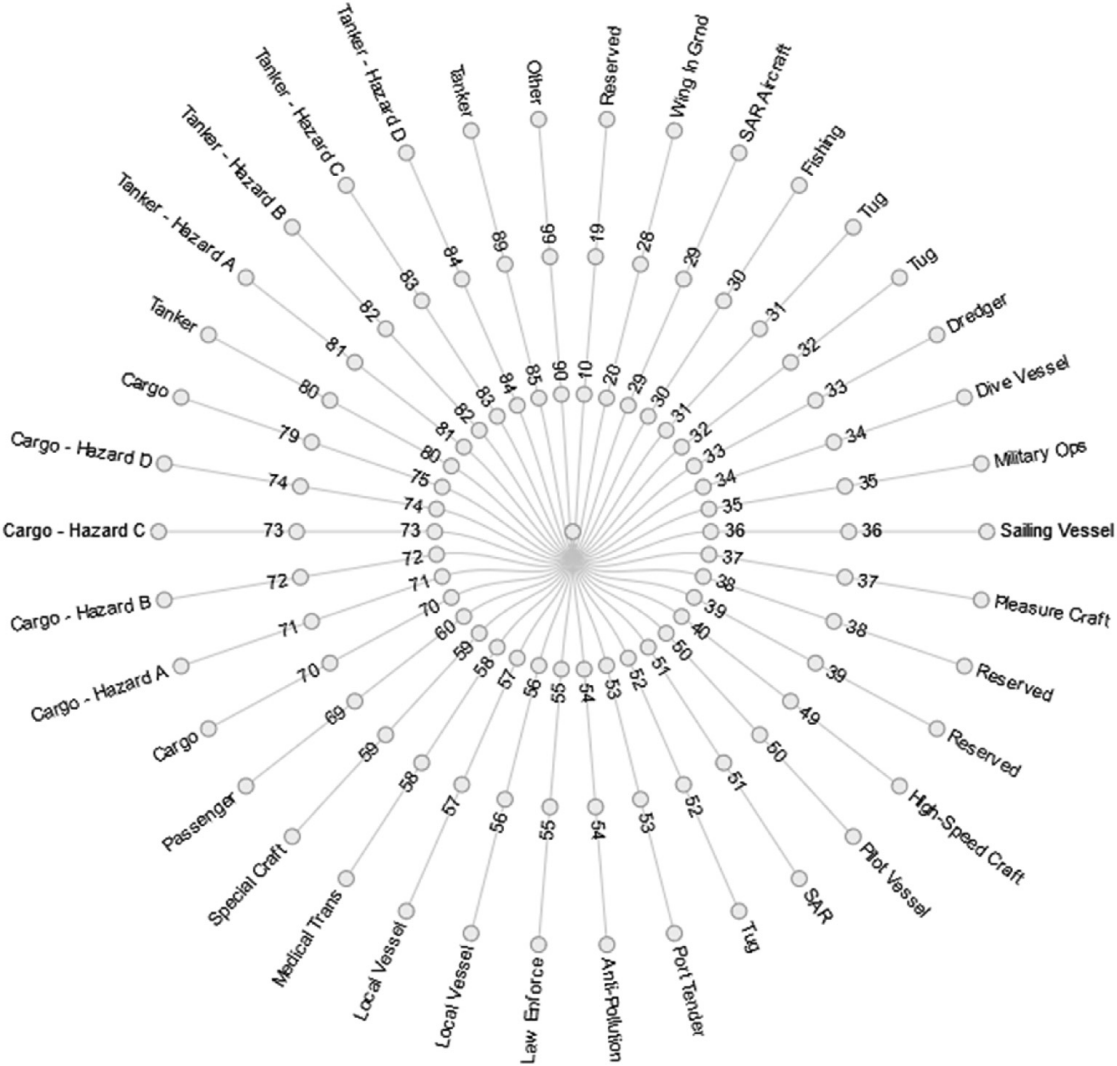
Circular representation of ship type data file (*A ship type is identified in AIS data by an integer in between values of inner and outer bounds*).

**Fig. 3:**
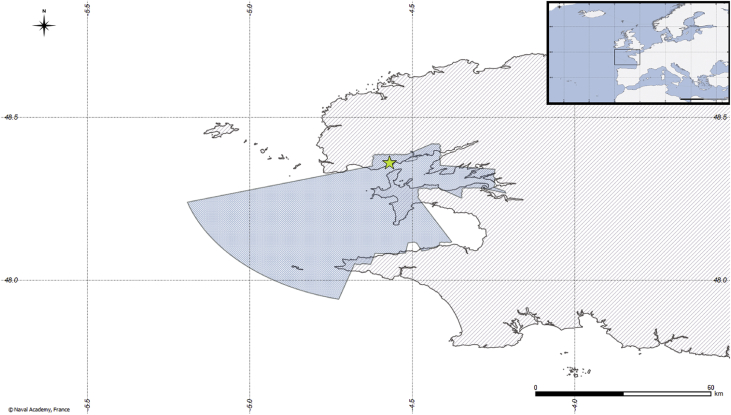
Terrestrial AIS receiver location and associated theoretical coverage.

**Fig. 4:**
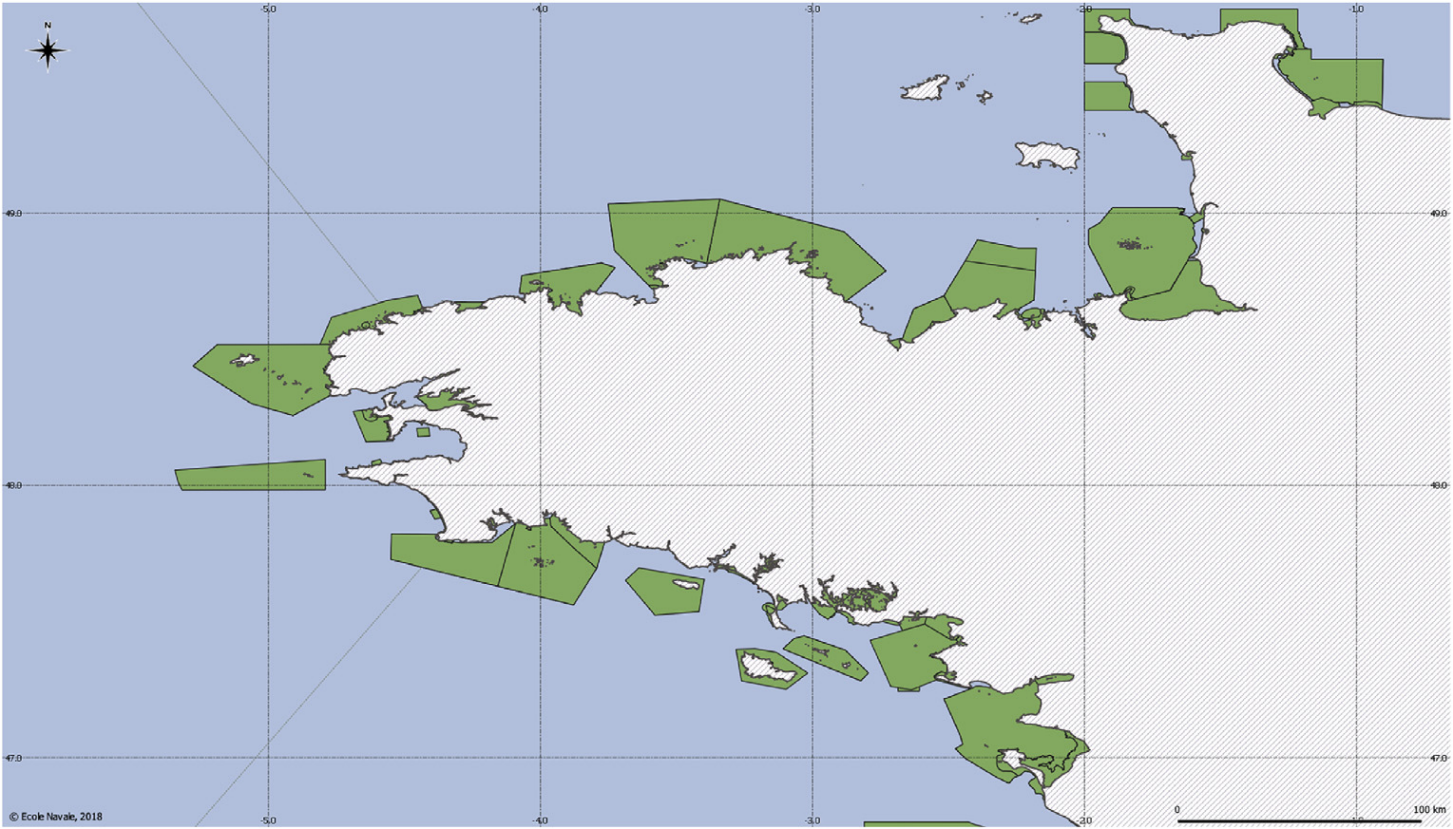
Natura 2000 areas (in *green*).

**Fig. 5:**
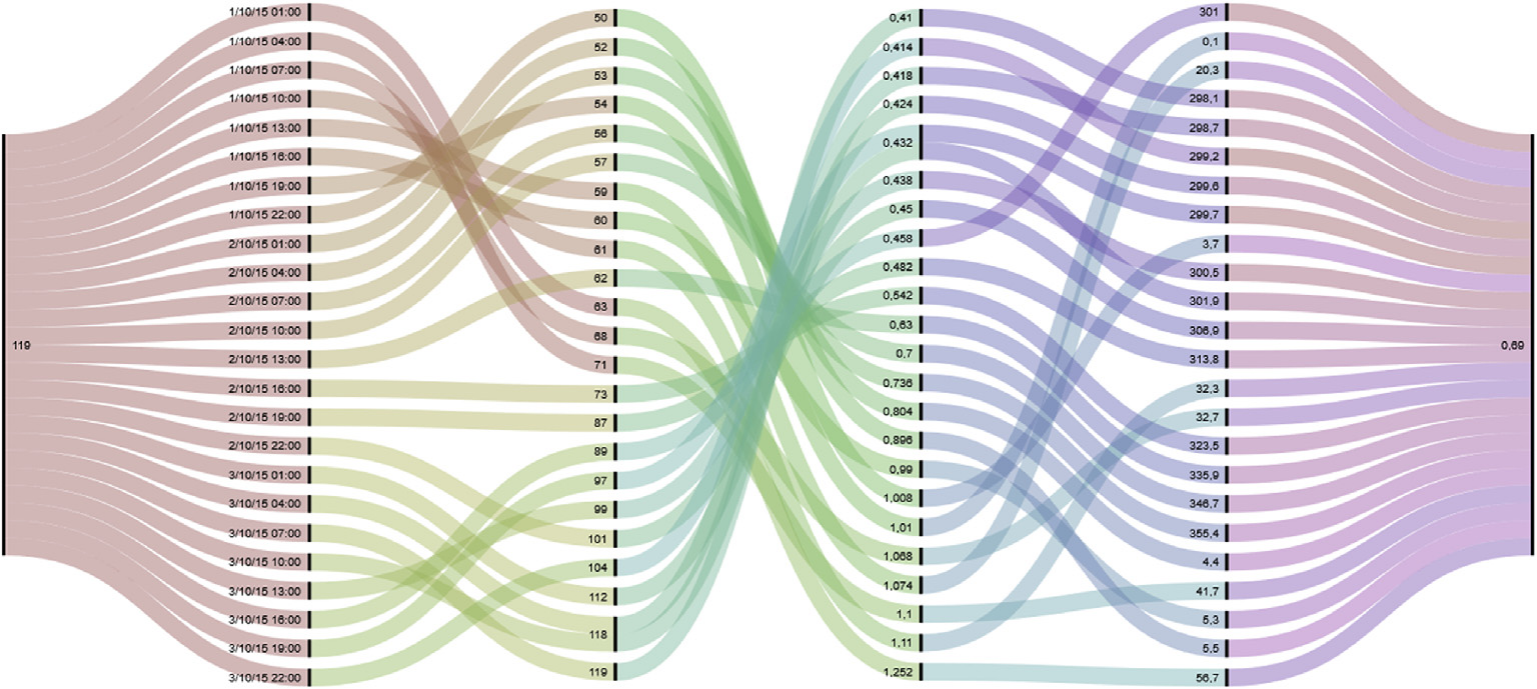
Alluvial diagram presenting ocean condition data file (*fields correlation along 3 days, from left to right: depth, date, mean wave length, height of wind and swell waves, wave mean direction, sea surface height*).

Fig. 1 illustrates the spatial coverage of the dataset: the dashed lines correspond to the spatial bounding box of the data,^2^ and the dark blue points represent the reported vessel positions. The location of the terrestrial AIS receiver is shown in Fig. 3, together with its associated theoretical coverage. Other navigation-related data are AIS status, codes, and types. Ship type is illustrated in Fig. 2. The dataset includes also vessel-oriented data (i.e., vessel registry), geographic data (i.e., spatial layers useful to contextualise vessel positions), and environmental conditions. Fig. 4 illustrates an example of the maritime protected areas included in the dataset (namely, Natura 2000). Fig. 5 correlates oceans conditions parameters (i.e., water depth, date, mean wave length, height of wind and swell waves, wave mean direction, sea surface height) along 3 days.

The dataset is composed by 34 data files in Comma-Separated Values (CSV) format and 15 geographic layers (as ESRI Shapefiles format), each provided with detailed meta-information specifying: source and originator, licence, spatial reference identifier (SRID), spatial coverage, temporal range, volume, together with a structured description of the data fields and types. Table 1, Table 2, Table 3, Table 4 summarise these meta-information by data category. The dataset is proposed with integration scripts and instructions to facilitate its insertion in a PostgreSQL/PostGIS relational database.

**Table 1:** Summary of navigation-related data.

	Source	SRID	Coverage	Period	Volume	Licence	Format and description
AIS dynamic messages	Naval Academy, France	EPSG 4326 (WGS84)	Dataset Area	01.10.2105 to 31.03.2016	18.648.556 decoded messages (1.05 GB)	CC-BY-NC-SA-4.0	Flat file (CSV) containing ships dynamic messages (ITU 1, ITU 2, ITU 3, ITU 18, ITU 19)
AIS static messages	Naval Academy, France	/	Dataset Area	01.10.2105 to 31.03.2016	1.032.187 decoded messages (82.0 MB)	CC-BY-NC-SA-4.0	Flat file (CSV) file containing static messages (ITU 5, ITU 19, ITU 24)
AIS Aids To Navigation (ATON)	Naval Academy, France	EPSG 4326 (WGS84)	Dataset Area	01.10.2105 to 31/03/2016	499.194 decoded messages (29.3 MB)	CC-BY-NC-SA-4.0	Flat file (CSV) containing ATON dynamic messages (ITU 21)
AIS Search and Rescue (SAR)	Naval Academy, France	EPSG 4326 (WGS84)	Dataset Area	01.10.2105 to 31.03.2016	4.446 decoded messages (221 KB)	CC-BY-NC-SA-4.0	Flat file (CSV) containing SAR dynamic messages (ITU 9)
AIS status, codes, and types	Naval Academy, France	/	Dataset Area	01.10.2105 to 31.03.2016	600 different codes	CC-BY-NC-SA-4.0	Flat files (CSV) containing meta information explaining navigational status, country codes, ship types, ATON
Receiver location	Naval Academy, France	EPSG 4326 (WGS84)	Dataset Area	01.10.2105 to 31.03.2016	1 point	CC-BY-NC-SA-4.0	ESRI Shapefile containing the position of the terrestrial receiver
Theoretical coverage	Naval Academy, France	EPSG 4326 (WGS84)	Dataset Area	01.10.2105 to 31.03.2016	1 polygon	CC-BY-NC-SA-4.0	ESRI Shapefile containing the polygon corresponding to the theoretical coverage of the AIS receiver

**Table 2:** Summary of vessel data.

	Source	SRID	Coverage	Period	Volume	Licence	Format and description
European Fishing Vessels Fleet Register	European Commission - Fisheries & Maritime Affairs	/	Europe	/ (last update in June 2017)	487.126 entries	Public	Flat files (CSV) containing the list of registered fishing vessels in Europe and their characteristics
French Fleet Register	French Frequencies Agency (ANFR) sourced by data.gouv.fr	/	France	/ (last update in June 2017)	180.817 entries	Open Licence	Flat file (CSV) containing the list of vessels registered by the French Frequencies agency (ANFR)

**Table 3:** Summary of geographic data.

	Source	SRID	Coverage	Period	Volume	Licence	Format and description
Ports of Brittany	Brittany region sourced by data.gouv.fr	EPSG 4326 (WGS84)	Brittany	/ (last update in Oct. 2017)	222 ports (190 KB)	Open Licence	ESRI Shapefile containing coordinates of ports of Brittany
Seadatanet Port Index	SeaDataNet	EPSG 4326 (WGS84)	World	/ (last update in Nov. 2016)	4896 ports (7.6 MB)	SeaDataNet Data policy	ESRI Shapefile containing coordinates of fishing ports throughout the world
World Port Index	National Geospatial-Intelligence Agency	EPSG 4326 (WGS84)	World	/( last update in Feb. 2016)	3684 ports (62.4 MB)	Public	ESRI Shapefile containing coordinates of ports throughout the world
European Coastline	European Environment Agency	EPSG:3035 (ETRS89)	Europe	/ (last update in Jul. 2013)	1 polyline 1 polygon (96 MB)	EEA standard re-use policy	ESRI Shapefile containing the European Coastline (polylines and polygons)
European Maritime boundaries	European Environment Agency	EPSG 4326 (WGS84)	Europe	/ (last update in Mar. 2005)	137 polylines (2.46 MB)	EEA standard re-use policy	ESRI Shapefile containing maritime boundaries that include territorial waters, bi- or multi-lateral boundaries as well as contiguous and exclusive economic zones
IHO World Seas	VLIZ, Flanders Marine Institute sourced by MarineRegions.org	EPSG 4326 (WGS84)	World	/(last update in Oct. 2017)	101 polygons (142 MB)	Public	ESRI Shapefile containing names and polygon of world seas
World Exclusive Economic Zones (EEZ)	VLIZ, Flanders Marine Institute sourced by MarineRegions.org	EPSG 4326 (WGS84)	World	/(last update in Oct. 2016)	2.192 polylines and 281 polygons (180 MB)	Public	ESRI shapefiles containing Exclusive Economic Zones Boundaries (polygons and polylines). Areas beyond this boundary can be classified as “High Seas"
FAO Fishing Areas	Food and Agriculture Organization of the United Nation (FAO)	EPSG 4326 (WGS84)	World	/ (last update in Apr. 2014)	322 polygons (8.81 MB)	Public	ESRI Shapefile containing fishing areas provided for statistics by the Food and Agriculture Organization
Estimated Fishing Areas	European Commission, ISPRA Joint Research Centre	EPSG 4326 (WGS84)	Dataset Area	01/09/2015 to 01/09/2015	502 polygons (7.01 MB)	Public	ESRI Shapefile containing estimated fishing areas made by European commission
Fishing Constraints	Naval Academy, France	EPSG 4326 (WGS84)	Dataset Area	05/02/2015 to 21/01/2016	2 polygons (29 KB)	Public	ESRI Shapefile containing two geographic areas where shellfish fishing activity is forbidden
Natura 2000 Areas	European Commission, Unit Nature & Biodiversity, Directorate-General Environment	EPSG:3035 (ETRS89)	Europe	2015	27.295 polygons (1.13 GB)	EEA standard re-use policy	ESRI Shapefile and flat files (CSV) containing geographic and descriptive data of European ecological sites

## 2 Experimental design, materials and methods

The following sections first provide a description of the four categories of data that constitute the dataset and how they have been collected and prepared: navigation data, vessel-oriented data, geographic data, and environmental data. Secondly the paper explains the experimental process and concludes presenting the broad impact of the dataset.

### 2.1 Navigation-related data

The AIS communicates 27 kinds of messages, each one having its own purpose in information transmission (positioning, nominative information, management …). The messages are broadcast in a theoretical range of circa 40 nautical miles. These AIS messages are binary messages that comply with the ITU-R.M 1371-5 [9] and NMEA 4.0 standards.^3^ In the dataset, two main classes of messages are considered: positioning and nominative information.

#### 2.1.1 Positioning and nominative information

*Positioning messages (ships’ dynamic messages):* Several AIS messages provide vessel positions that are acquired automatically by AIS transponders using embedded sensors (typically, GPS, gyroscope, loch, compass). To build the dataset, the ITU message types ITU 1, ITU 2, ITU 3, ITU 18, and ITU 19 were selected from which the following fields have been extracted:

• The Maritime Mobile Service Identity (MMSI) which is an international and unique ship identifier;
• The coordinates (longitude and latitude expressed using WGS84 reference system);
• The associated Speed Over Ground (SOG) in knots;
• The true heading in degrees (relative to true north) and the associated Course Over Ground (COG) also in degrees (direction of motion);
• The rate of turn, when available, expressed in degrees per minute;
• The navigational status, an integer encoding the current motion status of the ship (e.g. anchored, on the way sailing …).

These messages constitute an ordered time series. However, AIS messages do not embed the timestamp of the emission. Therefore, each message has been timestamped, with an integer corresponding to a UNIX epoch time^4^ upon reception by the receiving workstation (cf. Section 2.5). Fig. 1 illustrates the content of positioning messages reported on a map. The dashed lines correspond to the bounding box of data.

*Nominative messages (ships' static messages):* Several message types provide ship meta-information such as the ship name or voyage-related information. Some of these data are fully static (*i.e.* set at the initialisation of the AIS device onboard) and will not change during ship's life (e.g., ship dimension), while few others can evolve (e.g., the name can change depending on the owner) with varying frequency (e.g. destination should be updated at each different voyage). These fields are manually set and thus subject to errors, imprecisions or simply not fed. Nominative information collected for this dataset contains:

• The MMSI, and the ship identification number (an integer) provided by the International Maritime Organization (IMO);
• The international radio call sign (a string of characters);
• The name of the vessel (a string of characters) and associated ship type encoded with an integer;
• The reference point for reported position (biggest ships can reach 400 m length) and overall dimensions of the ship expressed with four integers describing a rectangle based on the reference point.

In addition, voyage-related information contains:

• The destination (next port of call) of the current trip (a string of characters manually entered) and associated Estimated Time of Arrival (ETA) expressed in format month-day-hour-minute (using the Coordinated Universal Time with time zone);
• The draught of the ship for the current voyage (expressed in meters by a real number between 0.1 and 25.5).

These fields have been extracted from ITU messages of types ITU 5, ITU 19, and ITU 24. Similarly to dynamic messages, each message has been timestamped with UNIX epoch time at the receiving workstation.

*Positioning messages (search and rescue dynamic messages):* Several *Search And Rescue* (SAR) aircrafts operating at sea are also equipped with AIS transceivers. These particular emitters provide their locations using message type ITU 9. The information extracted from SAR messages contains:

• The MMSI;
• The coordinates (longitude and latitude expressed using WGS84 reference system);
• The associated speed over ground in knots;
• The course over ground expressed in degrees (with respect to the true north);
• The altitude of the search and rescue aircraft (an integer ranging from 0 to 4094 m).

Each message has been also timestamped with UNIX epoch time at the receiving workstation.

*Positioning messages (aids to navigation dynamic messages):* Dynamic data of the AIS also include locations of *Aids To Navigation* (AtoN), typically buoys and lighthouses. An aid to navigation can be physical or virtual. Virtual AtoN do not exist physically and can be useful in time-critical situations and in marking/delineating dynamic locations or areas where navigational conditions change regularly. This equipment communicates information though the message type ITU 21. Information extracted from AtoN messages contains:

• The MMSI;
• The type of the aid to navigation encoded with an integer and associated name (a string of characters);
• The coordinates (longitude and latitude expressed using WGS84 reference system);
• The nature of AtoN (a Boolean value to distinguish between virtual and physical AtoNs).

Each message has been also timestamped with UNIX epoch time at the receiving workstation.

#### 2.1.2 AIS status, codes and types

Some data fields of AIS messages are encoded, usually with integer codes. To facilitate the understanding and the analysis of AIS data, these enumerations and the associated information have been included in the dataset, in Comma-Separated Values (CSV) files.

*Status:* The navigational status provided by positioning messages is encoded by an integer corresponding to 16 different status which names are themselves described by predefined strings of characters (e.g. moored, under way).

*Country Codes:* Each ship is registered in a country (*flag*). The country of each ship is encoded in the first three digits of each MMSI number (e.g., ‘227’ is used for France). Country codes and names (string of characters) are included in the dataset.

*Ship Types:* The ship type is encoded by an integer (Fig. 2) in nominative messages (e.g., ‘30’ is the code used for fishing vessels). The correspondence between 38 codes (integer) and the ship types (string of characters) as described in Ref. [9] is provided in this dataset. Additionally, a list of 233 refined ship types has been provided for advanced classification (this extended list is based on a commercial service's list^5^).

*AToN:* The type of aid to navigation (e.g., floating vs. fixed buoy, light, beacon) is encoded by an integer in the ITU 21 dynamic messages. This CSV data file provides a textual description (nature and type) for these codes.

#### 2.1.3 AIS receiver

*Receptor location:* The AIS messages have been collected using a single terrestrial receiver. The position of this receiver is given in the dataset and displayed by the yellow star in Fig. 3. It is a two-dimensional geometry of type *point* (*i.e.*, its altitude is at sea level) with coordinates (longitude and latitude) expressed using the WGS84 reference system.

*Theoretical coverage of the receiver:* Each terrestrial AIS receiver has a theoretical coverage that depends on its location and the surrounding topography.^6^ The theoretical coverage of the AIS receiver is given as a geometrical *polygon* with coordinates (longitude and latitude) expressed using the WGS84 reference system (the light blue polygon in Fig. 3). The receiver theoretical coverage has been calculated using the definition of sea areas from IMO resolution A801(19). It computes a circle of radius R nautical miles where R is equal to the transmission distance between a ship's VHF antenna at a height of 4 m above the sea level and the VHF antenna of the coastal station (at a height of H meters) which lies at the centre of the circle. The coverage has been computed with an antenna at 70 m above the sea level (66 m being the altitude of the location, plus four additional meters for the height of the building where the antenna is located).

### 2.2 Vessel data

Other nominative vessel information is available in official registers. For the vessels navigating in the area covered by the dataset, two of the most relevant institutional registers are included in this dataset.

#### 2.2.1 Community Fishing Fleet Register (European Commission)^7^

The European Commission freely provides a list of all fishing vessels that fly a flag of one of the countries of the Union. This fleet register concerns fishing vessels only which represent a fraction of the navigating vessels. The data fields that can be matched with AIS data (*i.e.*, same field either present or inferable within both datasets) are the international reference call sign, the name, and the vessel length. The fleet register also includes few technical details like length, gear type (61 different types), year of construction and engine power of the vessels.

#### 2.2.2 Civilian ships registered by ANFR (Agence Nationale des Fréquences)^8^

The fleet register provided by the French Frequencies Agency (ANFR) gathers a large number of French-registered vessels. The data fields include several normative information (MMSI, IMO numbers, registration numbers, ship name) that can be matched with AIS fields. The dataset also provides characteristics of the ship (type, length, and tonnage). Communication facilities (e.g. VHF) and information about ship licence (active, not active, and associated dates) are also described.

### 2.3 Geographic data

Geographic data provide complementary information to vessel movement data about topographic or regulatory context of vessel navigation.

#### 2.3.1 Ports

A lot of ships (e.g., tankers, cargos, passengers, ferries) have origins and destinations corresponding to ports. Having official lists of ports is therefore crucial. It can be used for instance to disambiguate the *Destination* field of AIS message 5 (filled manually). The dataset encompasses a detailed list of local ports and two worldwide lists (passing traffic around Brittany include world destinations).

*Ports of Brittany*.^9^ This dataset proposed by the Brittany region gathers the location of 222 ports around Brittany with names and coordinates expressed in the WGS84 system. A French law transferred, between 2015 and 2017, the management of ports from the region to departments. The dataset has been modified accordingly. In^9^, only ports still belonging to Britany region are proposed, however two of the four departments of Britany propose equivalent datasets,^10^^,^^11^.

*World Port Index (National Geospatial-intelligence Agency)*.^12^ The World Port Index (WPI) is a publication of the National Geospatial-intelligence Agency, which contains location and physical characteristics of, and the facilities and services offered by major ports and terminals world-wide. About of 3700 ports throughout the world are included in this dataset.

*SeaDataNet Ports Gazetteer*.^13^ This dataset provided by EMODnet, the Pan-European Infrastructure for Ocean and Marine Data Management^14^ focuses on halieutic ports. It contains names and coordinates of almost 5000 fishing ports throughout the world.

#### 2.3.2 European coastline (European Environmental Agency)^15^

The knowledge of the coastline is essential for the understanding of maritime movements. This dataset is a high resolution (1:100,000 scale) coastline of European shores (polylines and polygons, as shapefile), created by the European Environmental Agency (EEA) enabling highly detailed spatial analysis, such as the assessment of the proximity of vessels to coastlines or islands in support to maritime safety. It is a data derived from two sources: EU-Hydro^16^ and the Global Self-consistent, Hierarchical, High-resolution Geography Database (GSHHG).^17^

#### 2.3.3 Sea areas (International Hydrographic Organization)^18^

The dataset contains the main seas of the world (101) as polygons representing contiguous bodies of water, regionalised and divided into maritime basins. It can be useful to determine if two vessels are in the same region of the world. Areas covered (polygons concerned) by the navigation data are the *Celtic sea*, the *North Atlantic Ocean*, the *English Channel* and the *Bay of Biscay*.

#### 2.3.4 FAO Major Fishing Areas (Food and Agriculture Organization)^19^

This dataset contains the worldwide fishing regions established by the Food and Agriculture Organization (FAO) of the United Nations (UN). The boundaries were determined in consultation with international fishery agencies considering the distribution of the natural resources, national practices and boundaries, international conventions. It can be used for example to assess the declared provenance of fish against the areas in which a vessel effectively sailed or exhibited a fishing behaviour.

#### 2.3.5 Exclusive Economic Areas (Flanders Marine Institute)^20^

All the Exclusive Economic Zones (EEZs) of the world are gathered in this dataset. This can be useful to determine the quality of some at-sea operations as well as the competent court in some activities. Exclusive Economic Zones Boundaries are given as polygons and polylines. Areas beyond this boundary can be classified as *high seas*.

#### 2.3.6 European maritime boundaries (European Environment Agency)^21^

The dataset provided by the European Environmental Agency contains maritime boundaries in Europe that include territorial waters, bi- or multi-lateral boundaries as well as contiguous and exclusive economic zones (EEZs).

#### 2.3.7 Natura 2000 areas (European Environment Agency)^22^

The European database on Natura 2000 collects the reporting of the European protected areas, as an ecological network developed for the preservation of species and habitats (terrestrial and maritime areas). The dataset is managed by the European Environmental Agency and is built upon data submitted by European member states. The dataset contains descriptive data (e.g. the list of all species and habitat types) and spatial data (borders of sites). The version included in the dataset covers 2017 reporting. Fig. 4 shows areas of this data file.

#### 2.3.8 European fishing areas (European Commission Joint Research Centre)^23^

This dataset was created by the European Commission Joint Research Centre (JRC) and provides the fishing grounds in European waters, which can be used to assess the behaviour of fishing vessels. An assessment on the fishing pressure can also be extracted from this database. Fishing grounds have been derived from ship AIS positions collected along one year (from September 2014 until September 2015) and emitted by selected categories of fishing vessels exhibiting a fishing behaviour. Raw AIS data are originated from Volpe Centre of the U.S. Department of Transportation, the U.S. Navy, and MarineTraffic [2].

#### 2.3.9 Fishing constraints

This dataset contains two geographic areas where shellfish fishing activity is forbidden in the time window of the dataset.

### 2.4 Environmental data

Weather data and ocean data from forecast models and from observations (e.g., in-situ sensor data), which are openly available from several providers (see Table 4), can help validate analysis results and explain abnormal behaviour. For example, sea and weather conditions can force vessels to change direction or modify their normal route. They can also be used to characterise seasonal trends in traffic routes and to contextualise vessels kinematics such as speed.

**Table 4:** Summary of environmental data.

	Source	SRID	Coverage	Period	Volume	Licence	Format and description
Ocean conditions	SHOM, IFREMER	EPSG:4326 WGS84	Dataset Area	01.10.2105 to 31.03.2016	79.652.684 forecasts (1 per 3 hours) (3.02 GB)	Public	Flat files (CSV) containing the sea state forecast in the area, based on WAVEWATCH III model
Weather conditions	Met Office (United Kingdom) based on data provided by NOAA (United States) sourced by rp5.ua	EPSG:4326 WGS84	Dataset Area	01.10.2105 to 31.03.2016	71.516 observations (1 per hour) (5 MB)	Public	Flat files (CSV) containing observations from 16 coastal stations

#### 2.4.1 Ocean conditions

The ocean conditions were extracted from an hindcast database built with a WAVEWATCH III model [7] and provided by IFREMER.^24^ Initially built for the design of marine energy converters, it can also be used to study ships behaviour. Therefore, only the parameters relevant to this context were selected and stored in 6 CSV files (one file per month). The values are provided every 3 hours for each point of a regular spatial grid over the dataset bounding box. The grid spacing is 2 minutes, for both latitude and longitude. The parameters include coordinates (longitude and latitude expressed using the WGS84 reference system); bottom depth in meters; sea surface height above sea level in meters (tidal effect); significant height of wind and swell waves; mean wave length in meters; wave mean direction; and a timestamp expressed using epoch time (Fig. 5).

#### 2.4.2 Weather observations^25^

The coastal weather observations were recorded by 16 stations located in the south of England and along the French coasts. The data have been cleaned and formatted in the same way for the 16 selected stations, providing the most relevant fields such as temperature, atmospheric pressure, wind direction/speed and horizontal visibility, every hour over the 6 months period. The unformatted or potentially biased fields, especially those based on the human perception, have been removed.

### 2.5 Experimental process

#### 2.5.1 About coverage and time period

At the core of this heterogeneous dataset are AIS messages received by a terrestrial station located in the Brest roadstead, France (Fig. 3). This station is conveniently in direct sight of the roadstead bottleneck in order to cover the traffic in the roadstead, including the inbound, outbound movements and the traffic passing-by the Ushant Traffic Separation Scheme^26^ (TSS). In addition, local regulations from maritime authorities enforce fishing vessels to use AIS permanently, ensuring a rich-set of high-quality positional information [8]. The selected six-month period, from October 1st, 2015 to March 31st, 2016, is tied to fishing activities, and corresponds to shell fishing rights.

#### 2.5.2 AIS data

*Statistics.* The total number of messages received by the station is 24.033.893, from which 19.152.196 (79.7%) messages have been selected, to include vessels positions, aids to navigation and search and rescue. Among these selected messages, 1.032.187 are static messages (4.3%). 70.5% of vessels positions are located in a range of 10 km from the antenna. Although the theoretical maximum distance covered by the antenna is about 50 km (line of sight), the original dataset contains messages emitted at 700 km, due to the atmospheric ducting effect.

*Data cleaning.* AIS messages are well defined but sometimes erroneous because of system faults, data errors or deliberate manipulation. The dataset has been cleaned from errors resulting in 125.202 messages being discarded (0.5%) by the AIS parser (cf. next section). AIS management messages have been filtered out as well. Apart from errors and irrelevant messages, decoded AIS data are provided as received, including duplicates and other veracity issues that can be used to challenge the algorithms.

#### 2.5.3 AIS processing

*Raw messages.* Most AIS messages come without any timing information because the AIS has been initially designed as an anti-collision system to be used lively. The receiving station timestamps the messages immediately upon reception, in UTC format, and this timestamp is included in the navigation and nominative data. In a second step, the receiver broadcasts the timestamped messages, in the raw and unparsed format received to a central server which stores all the messages (*i.e.*, all the 27 different message types described in the ITU-R.M 1371-4 or NMEA 4.0 specification) in text files (one file per day).

*Processed messages.* The text files corresponding to the AIS messages received during the selected time period (October 1st, 2015 to March 31st, 2016) have been parsed and decoded. The parser, written in Java, extends and adapts the CC-BY-NC-SA 3.0 parser *aismessages*.^27^ The additional functionalities developed include: the connection to the database, ingestion, and outputting of UDP (User Datagram Protocol) streams and files (used by the AIS), automatic folder import, data logger for unparsed messages, data export translated to the standardised TAG Block format.^28^

The parsed data have been stored in a spatio-temporal database to facilitate the next processing steps. The data processing uses the widespread and open source relational database management system PostgreSQL,^29^ and its geospatial extension PostGIS^30^ for the treatment of spatial features. The data model of the developed AIS database encompasses a single schema, containing one table per message type. The AIS data included in the dataset are extracted from this database. Two files, aggregating different message types, have been created, containing respectively ships’ positioning messages (messages ITU 1, ITU 2, ITU 3, ITU 18, and ITU 19) and nominative messages (messages ITU 5, ITU 19, and ITU 24). Two additional files containing respectively search and rescue messages (ITU 9) and aids to navigation messages (ITU 21) have been also extracted from the database. During the aggregation process, messages have been spatially filtered using the bounding box illustrated in Fig. 1. Only message fields with navigation information have been selected, excluding few technical fields.

#### 2.5.4 Other data

For complementary data to the terrestrial AIS dataset (*i.e.* Sections 2.2, 2.3, 2.4) we favoured public sources like European institutions and projects [1], including: SeaDataNet,^31^ Copernicus,^32^ IFREMER,^33^ the EU Science Hub^34^ and EMODnet.^35^ Several of these data sources have been processed (e.g. original 1462 ocean condition files [7] have been aggregated in 6 files, one per month) mainly to facilitate their use. Only fishing areas [2] have been modified to limit the coverage to the bounding box illustrated in Fig. 1.

Additionally, nautical charts can be very useful for the understanding of maritime navigation. IHO S-57 (and its revision S-100) is the current IHO standard for digital hydrographic data. It defines a data model including 159 geo-object classes and the data structure and format used to implement it. These charts cannot be shared, but a technical note describing useful S-57 nautical charts and objects, including the necessary scripts to process them, has been prepared and proposed in Ref. [10].

#### 2.5.5 Data integration

To facilitate the integration and exploitation of the dataset, the database model used to prepare the data, including the SQL queries and the scripts for data integration, is proposed as part of the dataset (readme file, Windows and Linux scripts and needed SQL scripts). This relies on the relational database management system PostgreSQL and its PostGIS extension.

### 2.6 Maritime dataset ecosystem

The dataset described in this paper has been carefully prepared and validated, checking for errors, in order to offer the research community a set of heterogeneous real data to challenge, test and validate their research developments. The dataset was prepared to answer specific research questions on data integration and processing within the activities of the datAcron H2020 project (datacron-project.eu) [4], [5], [6]. It supported the development and assessment of real-time detection and prediction of trajectories and complex events related to moving entities^36^ which also generated datasets. These are available online:

• G. Santipantakis, G. Vouros, A. Glenis, C. Doulkeridis, A. Vlachou. *Link Discovery on AIS and Contextual data sets.* Dataset Version 1. https://doi.org/10.5281/zenodo.2597641, Mar 2019
• M. Pitsikalis, A. Artikis. *Composite Maritime Events.* Dataset Version 0.1. https://doi.org/10.5281/zenodo.2557290, Feb 2019
• G. Santipantakis, G. Vouros, A. Glenis, C. Doulkeridis, A. Vlachou. *Contextual maritime data set (RDF triples).* Dataset Version 1. https://doi.org/10.5281/zenodo.2576584, Feb 2019
• G. Santipantakis, G. Vouros, A. Glenis, C. Doulkeridis, A. Vlachou. *RDF triples of raw and trajectory synopses over AIS kinematic messages in Brest*, Dataset Version 1. https://doi.org/10.5281/zenodo.2576152, Feb 2019
• K. Patroumpas, D. Spirelis, E. Chondrodima, H. Georgiou, P. Petrou, P. Tampakis, S. Sideridis, N. Pelekis, Y. Theodoridis. *Final dataset of Trajectory Synopses over AIS kinematic messages in Brest area (ver. 0.8).* Dataset Version 1. https://doi.org/10.5281/zenodo.2563256, March 2018

## Footnotes

2 The bounding box of the dataset has the following coordinates: Longitude between −10.00 and 0.00 and Latitude between 45.00 and 51.00.

3 NMEA Standard: nmea.org/content/nmea_standards/nmea_standards.asp (accessed May 2019).

4 Number of seconds since 1970/01/01.

5 Marine Traffic, What is the significance of AIS ship type number [online]. help.marinetraffic.com/hc/en-us/articles/205579997-What-is-the-significance-of-the-AIS-Shiptypenumber (accessed May 2019).

6 Let us note that the practical coverage of an AIS receiver evolves regularly, mainly depending on weather conditions [11]. Additionally, some AIS messages may be propagated by AIS repeaters. Both affect the practical coverage.

7 Community Fishing Fleet Register, Fleet Register On the Net [online]. ec.europa.eu/fisheries/fleet/index.cfm (accessed May 2019).

8 Données radio maritime [online]. data.gouv.fr/fr/datasets/donnees-radio-maritime (accessed May 2019).

9 Région Bretagne, Ports de Bretagne [online]. data.gouv.fr/fr/datasets/ports-appartenant-a-la-region-bretagne (accessed May 2019).

10 Ports des Côtes-d'Armor [online]. data.gouv.fr/fr/datasets/ports-departementaux-des-cotes-darmor (accessed May 2019).

11 Ports du Morbihan [online]. data.gouv.fr/fr/datasets/ports-du-morbihan(accessed May 2019).

12 National Geospatial-intelligence Agency, World Port Index [online]. msi.nga.mil/NGAPortal/MSI.portal?_nfpb=true&_pageLabel=msi_portal_page_62&pubCode=0015^(^accessed May 2019^)^

13 SeaDataNet Ports Gazetteer [online]. seadatanet.maris2.nl/v_bodc_vocab_v2/welcome.asp (accessed May 2019).

14 EMODnet, the European Marine Observation and Data Network [online]. emodnet.eu (accessed May 2019).

15 European Environmental Agency, Europe coastline [online]. https://www.eea.europa.eu/data-and-maps/data/eea-coastline-for-analysis-1/gis-data/europe-coastline-shapefile/#permalinks-1 (accessed May 2019).

16 EU-Hydro [online]. land.copernicus.eu/imagery-in-situ/eu-hydro (accessed May 2019).

17 National Oceanographic and Atmospheric Administration, Global Self-consistent, Hierarchical, High-resolution Geography Database (GSHHG) [online]. ngdc.noaa.gov/mgg/shorelines (accessed May 2019).

18 International Hydrographic Organization, IHO Marine Regions [online]. marineregions.org/downloads.php (accessed May 2019).

19 FAO Major Fishing Areas [online]. fao.org/fishery/area/search/en (accessed May 2019).

20 Flanders Marine Institute, Exclusive Economic Areas v10 [online]. marineregions.org/downloads.php (accessed May 2019).

21 Maritime Boundaries [online]. eea.europa.eu/data-and-maps/data/maritime-boundaries (accessed May 2019).

22 Natura 2000 data - the European network of protected sites [online]. natura2000.eea.europa.eu. Data available at eea.europa.eu/data-and-maps/data/natura-9 (accessed May 2019).

23 European Commission Joint Research Centre (JRC): AIS derived high resolution fishing effort layer for European trawlers of more than 15 m long 2014–2015 [online]. data.jrc.ec.europa.eu/dataset/jrc-fad-ais1415 (accessed May 2019).

24 Institut français de recherche pour l'exploitation de la mer (IFREMER) [online]. ifremer.fr (accessed May 2019).

25 Weather history and forecast [online]. rp5.ua/archive.php (accessed May 2019).

26 A traffic separation zone is a zone separating the lanes in which ships are proceeding in opposite or nearly opposite directions; or separating traffic lanes designated for particular classes of ships proceeding in the same direction (IMO Ships Routing, 6th Edition).

27 AIS parser: github.com/tbsalling/aismessages (accessed May 2019).

28 NMEA Tag Blocks: catb.org/gpsd/AIVDM.html (accessed May 2019).

29 PostgreSQL data management system: postgresql.org (accessed May 2019).

30 PostGIS extension: postgis.net (accessed May 2019).

31 Seadatanet, Pan-European Infrastructure for Ocean and Marine Data Management [online]. seadatanet.org (accessed May 2019).

32 Copernicus marine environment monitoring service [online]. marine.copernicus.eu (accessed May 2019).

33 Institut français de recherche pour l'exploitation de la mer (IFREMER) [online]. ifremer.fr (accessed May 2019).

34 The European Commission's science and knowledge service EU Science Hub ec.europa.eu/jrc/en (accessed May 2019).

35 EMODnet, the European Marine Observation and Data Network [online]. emodnet.eu (accessed May 2019).

36 Since its creation on February 2018, the dataset (or part of it) has been downloaded in average 217 times per month (update on the 01/12/2018).

## Footnotes

1 Brest roadstead in which the AIS receiver is located has a local regulation enforcing fishing vessels to use AIS permanently, thus providing high-quality positional information.
